# Step-by-step Laparoscopic Vesiculectomy for Hemospermia

**DOI:** 10.1590/S1677-5538.IBJU.2016.0127

**Published:** 2017

**Authors:** Marcos Figueiredo Mello, Hiury Silva Andrade, Victor Srougi, Marco Antonio Arap, Anuar Ibrahim Mitre, Ricardo Jordão Duarte, Miguel Srougi

**Affiliations:** 1 Divisão de Urologia do Departamento de Cirurgia, Universidade de São Paulo, SP, Brasil

## Abstract

Hemospermia has been considered as a benign and self-limiting condition. It usually has an inflammatory or infectious cause. However, recurrent or persistent hemospermia may indicate a more serious underlying pathology, especially over 40 years of age. Biopsy or surgical excision is indicated in cases of suspicious findings during investigation, such as cysts or masses. Open surgery has been considered the definitive form of treatment, however, it can be associated with significant morbidity. With growing experience in laparoscopics, this approach is becoming the preferable way to access the seminal vesicles. Our objective is to demonstrate a step-by-step operative technique for laparoscopic unilateral vesiculectomy approach in a man with hemospermia.

**Case:**

A 61 year-old man presented with 1 year of hemospermia. He was treated empirically with a fluoroquinolone plus a nonsteroidal anti-inflammatory without resolution of symptoms. Ultrasonography and MRI showed a solid-cystic mass in the right seminal vesicle. The patient was submitted to a laparoscopic unilateral vesiculectomy. Histopathological analysis showed intraluminal dilatation with blood content. During follow-up, complete resolution of symptoms was seen.

**Results:**

Three patients composed our cohort. Mean age was 53 years-old (range 45-61 years), the right side was more commonly affected (two unilateral on the right and bilateral). Mean operative time was 55 minutes (range 40-120min). One patient presented amyloidosis in the histopathological analysis. All cases presented complete resolution of symptoms.

**Conclusions:**

Laparoscopic vesiculectomy is a safe and feasible approach in cases of hemospermia. This technique showed good outcomes and minimal morbidity.

## ARTICLE INFO

**Available at: http://www.intbrazjurol.com.br/video-section/20160127_mello_et_al/**

**Int Braz J Urol. 2017; 43 (Video #12): -783**

